# Hereditary Leiomyomatosis and Renal Cell Cancer-Recognizing Patterns May Save Lives

**DOI:** 10.15586/jkcvhl.v9i2.222

**Published:** 2022-05-26

**Authors:** Tavares Catarina, Maria Sofia Quental, José Ricardo Brandão, Miguel Silva-Ramos

**Affiliations:** 1 Centro Hospitalar Universitário do Porto, Largo do Prof. Abel Salazar, Porto, Portugal;; 2 Instituto de Patologia e Imunologia Molecular da Universidade do Porto (IPATIMUP), Rua Júlio Amaral de Carvalho 45, Porto, Portugal

**Keywords:** fumarate hydratase, hereditary renal cell cancer, HLRCC, renal cell carcinoma

## Abstract

In 2001, Finish investigators described a rare familiar syndrome characterized by an inherited susceptibility to cutaneous leiomyomas, uterine leiomyomas, and renal cell carcinoma (RCC). Hereditary leiomyomatosis and renal cell cancer (HLRCC) is now linked to a germline mutation in the *fumarate hydratase* (*FH*) gene that encodes a Krebs cycle enzyme, transforming fumarate to malate. The accumulation of fumarate, an oncometabolite, promotes tumorigenesis. We present a case of a 41-year-old female diagnosed with HLRCC after a radical nephrectomy due to renal cell cancer. Genetic analyses confirmed a novel *FH* mutation. Close follow-up allowed for a precocious diagnosis of a metachronous renal tumor and later a hepatic metastasis. Her family was also counseled and offered genetic testing. As observed in this case, the diagnosis of HLRCC is of paramount importance for patients and their families: there is a 15% cumulative lifetime risk of developing RCC, which frequently occurs in young patients and metastasizes at an early stage. Implementing a regular follow-up with adequate imaging examinations may help save lives.

## Introduction

In 1974, the association of cutaneous and uterine leiomyomas in some families was recognized as “Reed syndrome” (1). Almost 30 years later, in 2001, Finish investigators reported families in which cutaneous and uterine leiomyomas occurred in combination with renal carcinoma. This rare syndrome, known as hereditary leiomyomatosis and renal cell cancer (HLRCC) was linked to a germline mutation in the *fumarate hydratase* (*FH*) gene that encodes a Krebs cycle enzyme, which transforms fumarate to malate (2). HLRCC has since been reported in nearly 300 families worldwide (3, 4). The *FH* mutation is inherited in an autosomal dominant pattern and more than 200 different mutations have been described in the coding region of the gene. However, not all patients that inherit germline *FH* mutations develop tumors. In accordance with Knudson’s “two-hit” hypothesis, an additional acquired somatic mutation in the wild-type allele is necessary for the growth of tumors (although this has not been demonstrated in all cases) (5). The presence of both mutations leads to a marked reduction or total inactivation of fumarate hydratase enzyme. The accumulation of fumarate, an oncometabolite, promotes the development of *FH*-deficient tumors. However, the mechanism that leads to tumorigenesis in the presence of excessive fumarate has not been understood completely. A study conducted by Sciacovelli et al. advocates that fumarate gives way to epigenetic changes in microRNA, causing the expression of genes that initiates epithelial to mesenchymal transition. This phenotypic switch has been linked to cancer initiation, invasion, and metastasis (6). Another hypothesis is that disruption in Krebs cycle leads to a metabolic shift from oxidative phosphorylation to aerobic glycolysis for production of energy, and increasing lactate and reactive oxygen species. These metabolites lead to a state of pseudohypoxia through upregulation of hypoxia-inducible factor 1-alpha (HIF-1α) and HIF-2α, thereby promoting angiogenesis, cell proliferation, and, hence, tumorigenesis (7).

Hereditary leiomyomatosis and renal cell cancer can present in multiple forms, even in members of the same family. Cutaneous piloleiomyomas are the most common clinical feature in HLRCC, occurring in over 80% patients, with a higher penetrance in males (8). These tumors present as firm, skin-colored, or reddish-brown papules or nodules on the limbs and trunk in early adulthood (9). The lesions are frequently symptomatic, ranging from sensitivity to touch, pain with low temperature or paresthesia. However, some patients may only exhibit one or two discrete lesions, which may be asymptomatic. Consulting a dermatologist may help in cases where the urologist suspects of HLRCC (9).

Uterine fibromyomas are frequently large and symptomatic, occurring earlier than in the general population. Of the patients who present with uterine myomas, 70% are diagnosed prior to the age of 30 (9,10). Wei et al. found that 50% of women in their cohort who required surgery for fibromyomas had a myomectomy or hysterectomy at the age of 30 years or younger (11). Similarly, in a recent description of a Spanish cohort with 197 HLRCC patients, 55.4% of females had a hysterectomy at a median age of 34 years (4). These tumors are thus an important source of morbidity in women of child-bearing age.

Renal cell carcinoma (RCC) occurs in around 20% of affected families but has a very fluctuating prevalence (12). In most case series, tumors are solitary and unilateral (13). The average age at diagnosis is 41 years but has been described in some patients from 10 to 90 years of age (12). Sánchez-Heras et al. reported of a Spanish cohort, one of the largest to date, in which 10.9% of HLRCC patients presented with RCC at a median age of 37.4 years, including four patients diagnosed at an age of less than 25 years (4). Defining the patients that are at a risk for RCC has been challenging, as the type of *FH* mutation does not appear to be associated with the risk of developing RCC, and the individual risk does not appear to be higher in families with documented cases of RCC. Papillary type II has been a classical tumor associated with HLRCC but others have been described as well, such as tubulocystic carcinoma, collecting duct renal carcinoma, oncocytic, or even clear cell carcinoma (4). The updated 2016 World Health Organization (WHO) classification of tumors of the urinary system added a distinct entity of renal tumors associated with HLRCC because of their characteristic features—a papillary architecture with abundant eosinophilic cytoplasm, large nuclei, and very prominent nucleoli with perinucleolar clearing (14). Identifying such cases is crucial as these tumors are usually aggressive and metastasize early (9).

Diagnosing HLRCC is of paramount importance for patients and their families. Retrospective studies of families with HLRCC indicate a 15% cumulative lifetime risk of a patient developing this lethal form of RCC (9). The diagnosis allows for early screening of renal tumors, planning a family in young female patients, and genetic counseling of potentially affected family members, although there is no consensus regarding the frequency and method of screening.

We present a case of a young female patient with HLRCC. Her urologist suspected of this syndrome after observing her cutaneous leiomyomas and acknowledging her young age at presentation of renal cancer. The recognition of this pattern by her observant urologist may now allow her family members to be examined and closely followed to detect renal tumors precociously, as they represent a significant cause of mortality in such families.

## Case Report

**Figure 1:**
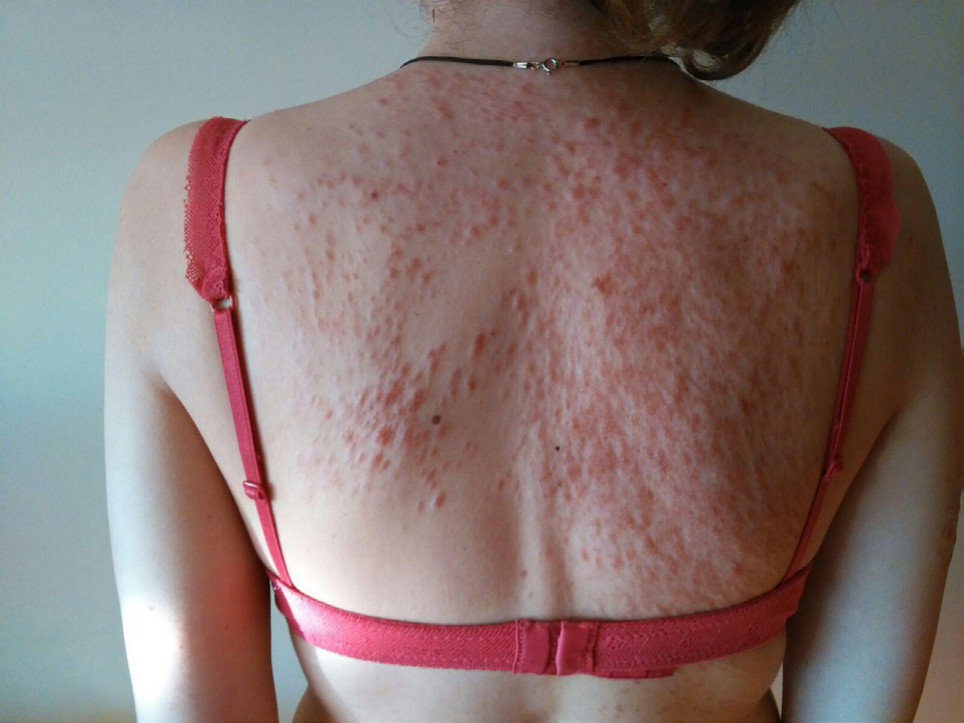
Cutaneous leiomyomas on patient’s back, leading to suspicion of hereditary leiomyomatosis and renal cell cancer (HLRCC).

**Figure 2:**
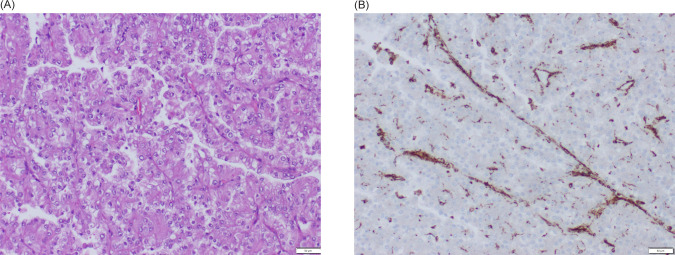
Pathology of RCC. (A) Neoplasm with papillary-predominant pattern, formed by clear cells with an irregular nucleus and a perinucleolar halo. (B) Immunohistochemistry with antibody for fumarate hydratase presents immunoreactivity only in stromal cells, while tumoral cells are negative.

**Figure 3:**
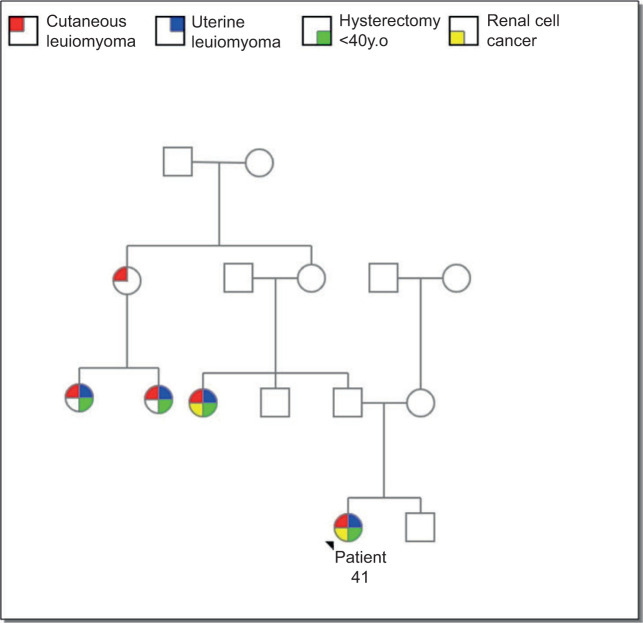
Pedigree of the patient.

In January 2016, a 41-year-old female presented to the emergency department with hematuria and right lumbar pain. She had a history of hypertension and a hysterectomy at the age of 38 because of a large symptomatic myoma. After multiple reddish-brown, painful skin nodules appeared on her arms, legs, and back, she had a skin biopsy, which confirmed piloleiomyomas (Figure 1). Her blood tests indicated slight anemia, and urine analysis confirmed macroscopic hematuria. A computed tomography (CT) scan demonstrated three contrast-enhancing renal tumors of the right kidney, with diameters of 4.9, 4.8, and 1.7 cm (Figure 2). No evidence of metastatic disease was observed in subsequent CT staging examinations. A radical right laparoscopic nephrectomy was ensued in February 2016. The pathology examination revealed three renal tumors as papillary type II RCC.Considering her previous medical conditions and after observing her cutaneous lesions, her attending urologist suspected of HLRCC. The patient’s family history also included a brother and other relatives having cutaneous leiomyomas (Figure 3). Her paternal aunt, treated at another institution, had had a nephrectomy because of papillary type II RCC as well as an early hysterectomy and biopsy for cutaneous leiomyomas. The patient received genetic testing, which revealed a heterozygous variant (c.322C>T [p. Gln108^*^]) of the *FH* locus, a variant not reported previously in literature but already classified as pathogenic or potentially pathogenic in ClinVar database. This variant predictably leads to production of a truncated inactive enzyme or even none in the least.

After nephrectomy, the patient was followed for every 6 months with CT scans. Her family members were contacted and informed about the identified syndrome to be able to perform a genetic test, if desired. In February 2019, an isolated hepatic metastasis was identified and the patient had a laparoscopic metastasectomy. Her last CT scan conducted in September 2020 indicated no evidence of malignant disease.

## Discussion

The patient in our clinical case presented with three manifestations of HLRCC, although this does not take place always. As such, urologists must be familiarized with the clinical features of HLRCC and pay attention to patient’s family history to suspect patient’s affectedness. Clinical diagnostic criteria were suggested by Smit et al., which when present must lead to genetic testing for final confirmation of the syndrome (10). Histopathological confirmation of multiple cutaneous piloleiomyomas is considered a major criterion, while treatment of symptomatic uterine myomas in women aged less than 40 years, papillary type II RCC prior to the age of 40, or first degree relative with one of the aforementioned conditions is considered minor criterion. Patient with a major criterion is considered as “likely to have HLRCC,” while HLRCC must be suspected if the patient presents with two or more minor criteria.

Genetic testing is mandatory for confirmation of HLRCC. *FH* gene, located on chromosome 1q42, is demonstrated to have germline mutation in 90% of patients with HLRCC: multiple different mutations have been described from missense to frameshift, nonsense, splicing, and deletion variants, translating into a nonfunctioning or absent protein. In this case, the encountered c.322C>T (p. Gln108^*^) variant probably leads to the production of a truncated protein or total absence of protein because of the degradation of transcript by nonsense-mediated mRNA decay. Following the guidelines of the American College of Medical Genetics and Genomics (ACMG), the variant was classified as “likely pathogenic” (15). No correlation was established between the location or type of mutation and the development of kidney cancer in HLRCC, as observed in other forms of hereditary kidney cancer such as von Hippel-Lindau (VHL) disorder (16). Therefore, all family members who inherit the mutation must be considered at a risk for occurrence of RCC.

After confirming the diagnosis of HLRCC, the patient was counseled and followed up with thoraco-abdominal CT scans every 6 months. No formal guidelines on screening have been formulated; different options have been suggested in literature, varying from ultrasound to tomography or magnetic resonance imaging (MRI), starting in childhood with a frequency of 6 months to a year. The Second Symposium on Hereditary Leiomyomatosis and Renal Cancer held in Paris in 2013 recommended annual MRI (preferred) or CT scan, with/without contrast, starting at the age of 8–10 years. The same recommendations are stated in the 2020 National Comprehensive Cancer Network^®^ (NCCN) Guidelines (17). Ultrasonography alone is not recommended because the lesions may be isoechoic and not detectable. Implementing a regular follow-up with adequate imaging may help save lives by allowing an early diagnosis of these tumors.

A single liver metastasis was encountered after 3 years of partial nephrectomy and the patient had a hepatic metastasectomy. Treatment of metastatic renal cell cancer in HLRCC patients is a challenge, as traditional therapy has not been effective (18). As no specific therapies have been approved by the US Food and Drug Administration (FDA), the NCCN guidelines recommend erlotinib plus bevacizumab, as they have demonstrated benefits in a phase II trial (17). The trial established an overall response rate of 60% in patients treated with Erlotinib Plus Bevacizumab and a median progression-free survival of 24.2 months in 20 patients with HLRCC-associated RCC (19).

## Conclusion

In order to have the best available opportunities for HLRCC patients, dermatologists, gynecologists, and urologists must understand this complex syndrome and be aware of when to suspect it. The implementation of screening protocols for affected family members and improved follow-up of RCC patients must also be established. Patients with metastatic RCC must be enrolled in clinical trials whenever possible. A better understanding of the pathophysiology of these renal tumors is also important for the development of targeted therapies. For example, taking into account that HLRCC tumors depend on aerobic glycolysis because of dysregulation of Krebs cycle, the use of inhibitors of glucose transport or glycolysis may be an effective treatment option.
